# Measuring biological age using metabolomics

**DOI:** 10.18632/aging.104216

**Published:** 2020-11-29

**Authors:** Oliver Robinson, Chung-Ho E. Lau

**Affiliations:** 1 MRC Centre for Environment and Health, Department of Epidemiology and Biostatistics, School of Public Health, Imperial College London, London, UK

**Keywords:** biological age, metabolomics, metabolic, biomarkers, epigenetic

In a recent study, Robinson and colleagues [1] applied metabolomics to accurately predict the age of working-age participants of the British AIRWAVE occupational cohort. Metabolomics is the global profiling of small molecules (generally <1 kDa) or metabolites that are present in biological samples and is increasingly applied in population based epidemiological studies. The work is part of trend of applying new technologies, such as high throughput “omics” analyses, to answer old questions about why and how we age. Inspired by the widely used epigenetic clock of Horvath [2], the study used a similar statistical framework to show that chronological age may be predicted from blood and urine samples in independent testing sets with comparable accuracy to prediction models constructed from DNA methylation data. Although both data types can provide global read-outs of biological pathway activity, metabolomic and epigenetic data provide different challenges and advantages for the assessment of aging.

Prediction of chronological age itself is of limited use beyond specialised fields such as forensic science. However, the new field of Geroscience proposes that biological aging, a set of interrelated molecular and cellular changes associated with aging, drive the physiological deterioration that is the root of multiple age-related health conditions [3]. Robinson and colleagues [1] observed that having an older predicted metabolomic age than chronological age (“age acceleration”) was associated with multiple risk factors of premature mortality, suggesting the metabolomic model also captured differences in biological age. Furthermore, metabolic pathways enriched among the model predictors were related to proposed biological age hallmarks such as mitochondrial disfunction, intra-cellular signalling and nutrient sensing.

Somewhat surprisingly, age acceleration measured through metabolomics was uncorrelated with assessments based on established epigenetic clocks. One may speculate that epigenetic clocks specifically target the hallmark of epigenetic stability which was not captured through metabolomic analysis. Negligible correlations between different biological age markers have been reported in other cross-sectional studies possibly suggesting that the different hallmarks of aging are independent rather that multifaceted expressions of the same core process [4]. However, the various manifestations of aging may not occur simultaneously and lagged longitudinal analyses may be more appropriate to understand relationships between biological age markers [4]. At any rate, metabolomic aging assessment provides a useful new tool to complement other biological age markers.

Metabolomic age acceleration showed similar or somewhat stronger associations with most of the assessed risk factors as epigenetic clocks that were similarly trained on chronological age. However greater sensitivity to risk factors was observed with an epigenetic clock trained on “phenotypic age” a composite of clinical markers selected based on prediction of time-to-death. This raises important questions regarding the optimum approach to derive biological age markers using untargeted approaches such as “omics”. Aging is driven by both intrinsic (biological) aging and disease specific processes and it is practically difficult to separate these processes. Identifying molecular markers associated with chronological age, particularly in populations including younger, healthy individuals, presents one route to identifying intrinsic biological age markers, distinct from early effect markers of specific disease processes, that may precede subsequent changes in phenotype and health [4]. It is of interest that another recent study in older individuals identifying metabolomic predictors of the the frailty index, a measure of functional age, noted dysregulation of vitamin E and carnitine shuttle metabolic pathways [5]. These pathways were also observed to be associated with chronological age in a younger population by Robinson and colleagues [1].

Metabolomic analysis in blood can provide a more complete picture of biological processes involved in aging than DNA methylation analysis, as metabolites represent the final products of cellular metabolism including in organs and tissues throughout the body, rather than in just blood cells themselves. All the hallmarks of aging may be expected to have detectable effects on the metabolome and overlap significantly with the effects of metabolic disorders [6]. The metabolomic study of age is an active area with many important recent contributions [7], including the development of a searchable database of age-associated metabolites [8]. However large challenges in the field remain including comparability and reproducibility across studies, metabolome coverage and metabolite annotation. Furthermore, the metabolome is highly influenced by the environment, and future studies need to incorporate multiple populations and longitudinal sampling to minimise confounding by cohort effects, particularly in highly age-stratified populations.

Age-related disease including cancers, cardiovascular disease and dementia, provide the greatest health burden in developed countries and it is hoped that targeting the biological aging process, particularly earlier in life, will have greater success in reducing the burden of disease than the current approach of disease-specific treatments in later life [3]. Assessment of biological age through metabolomics present one step towards answering the key questions in Geroscience: What are the changes that occur from age 30 to age 70 to increase the chance of dying by roughly 30-fold; and what determines variability in aging?
